# Supplementary figures and images for: Comparing Bayesian and non-Bayesian accounts of human confidence reports
Source: PLoS Comput Biol. 2018 Nov 13;14(11):e1006572. doi: 10.1371/journal.pcbi.1006572 (PMC6258566; doi:10.1371/journal.pcbi.1006572)

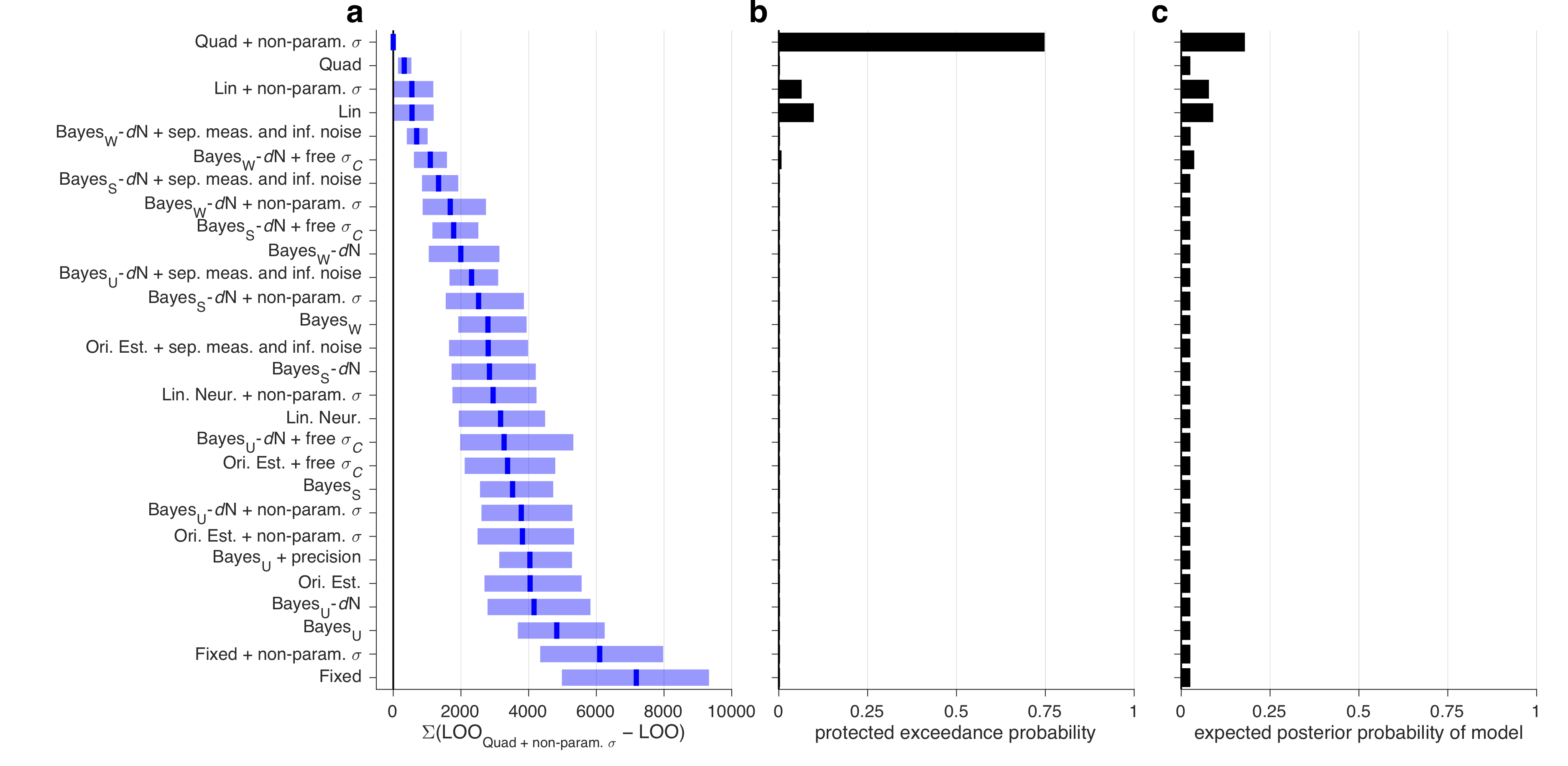

Supplement: S1 Fig — Models were fit jointly to Task A and B category and confidence responses. (a) Medians and 95% CI of bootstrapped sums of LOO differences, relative to the best model. Higher values indicate worse fits. (b) The protected exceedance probability, i.e., the posterior probability that a model occurs more frequently than the others [91]. (c) The expected posterior probability that a model generated the data of a randomly chosen subject [92]. (TIF) [file pcbi.1006572.s001.tif]

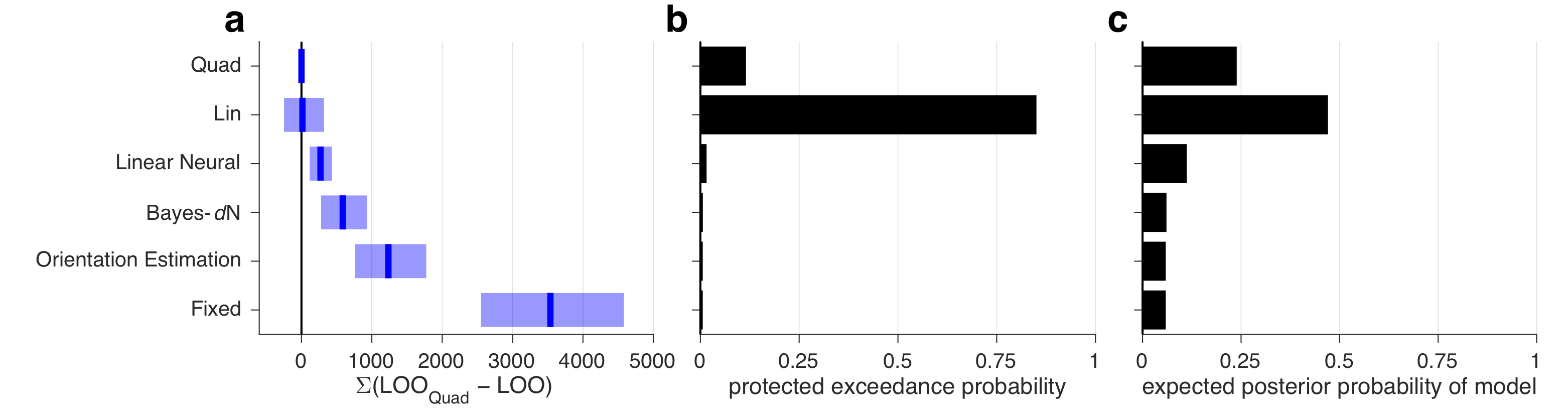

Supplement: S2 Fig — Models were fit to Task A category and confidence responses. See S1 Fig caption. (TIF) [file pcbi.1006572.s002.tif]

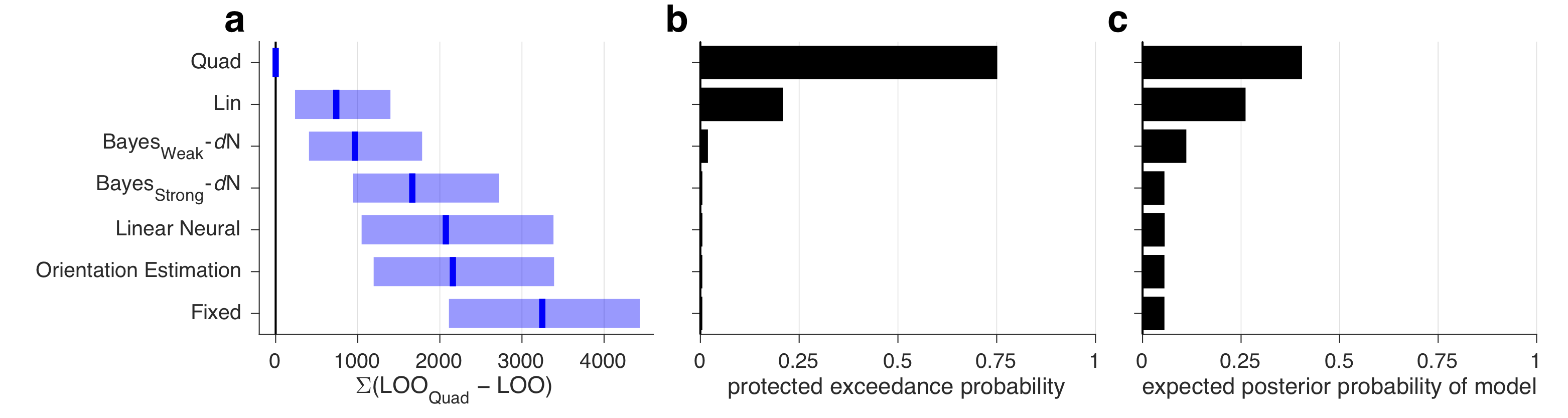

Supplement: S3 Fig — Models were fit to Task B category and confidence responses. See S1 Fig caption. (TIF) [file pcbi.1006572.s003.tif]

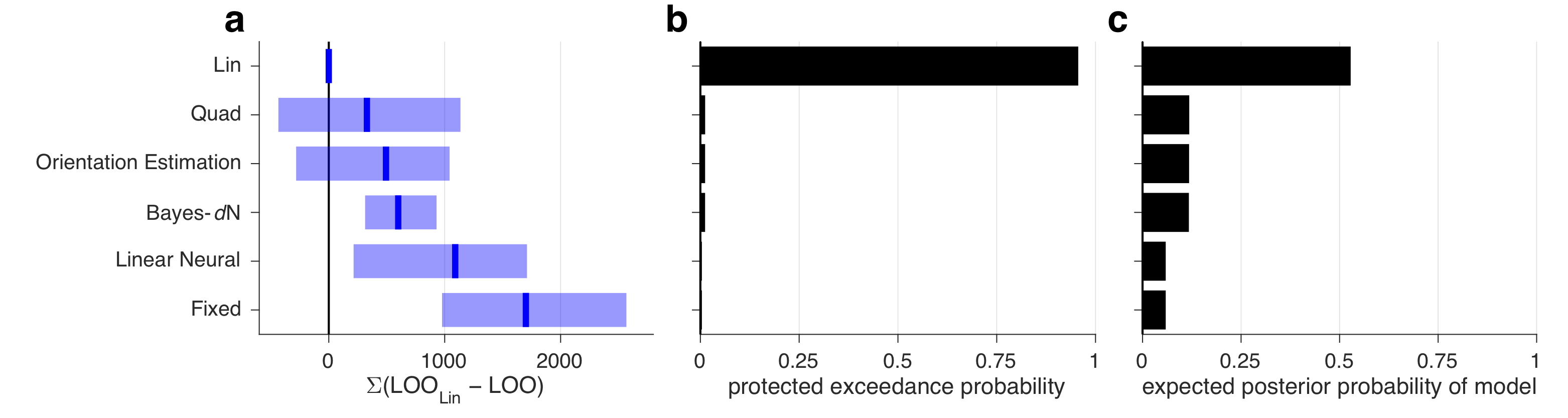

Supplement: S4 Fig — Models were fit jointly to Task A and B category choices. See S1 Fig caption. (TIF) [file pcbi.1006572.s004.tif]

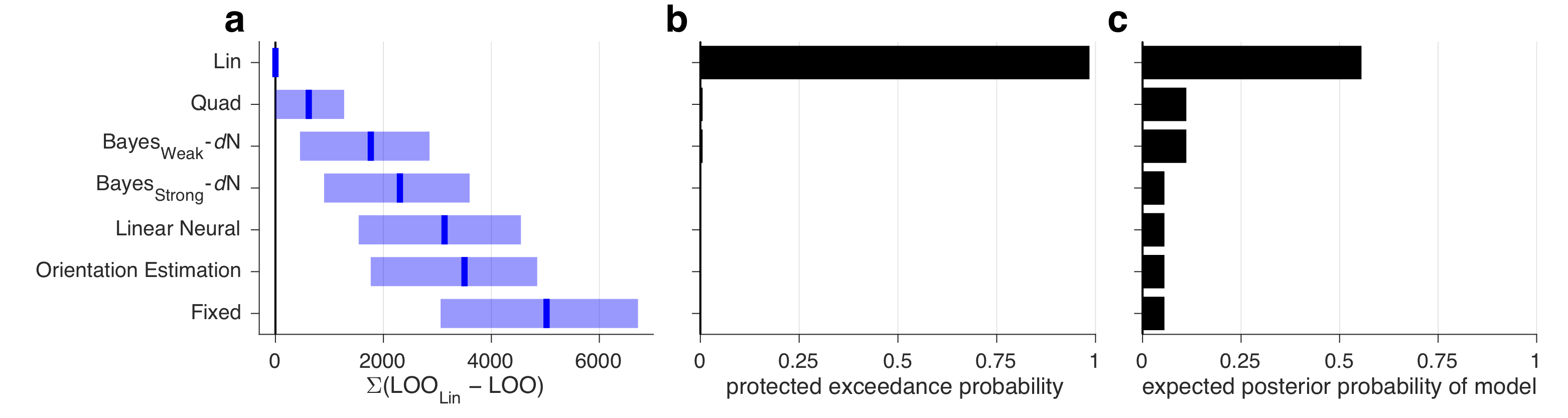

Supplement: S5 Fig — Noise parameters were fit to Task A category choices and then fixed during the fitting of Task B category and confidence responses. See S1 Fig caption. (TIF) [file pcbi.1006572.s005.tif]

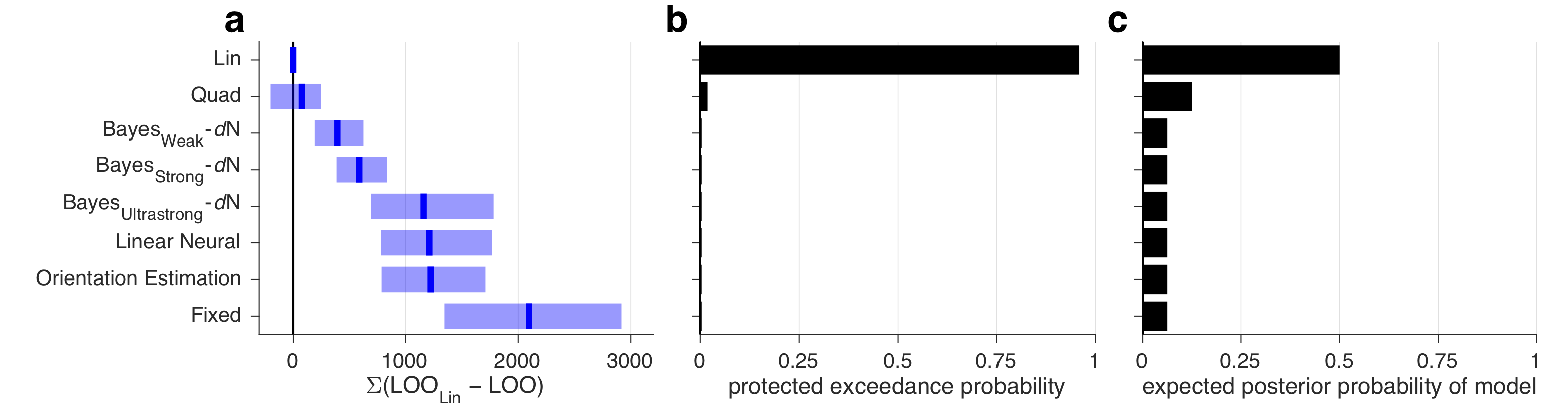

Supplement: S6 Fig — Models were fit jointly to Task A and B category and confidence responses. See S1 Fig caption. (TIF) [file pcbi.1006572.s006.tif]

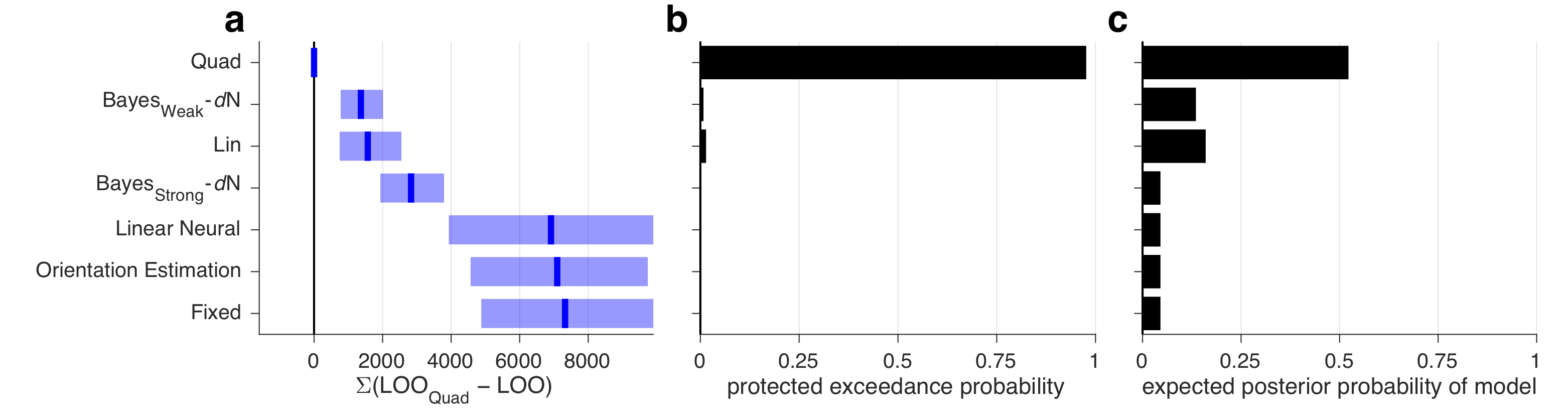

Supplement: S7 Fig — Models were fit to Task B category and confidence responses. See S1 Fig caption. (TIF) [file pcbi.1006572.s007.tif]

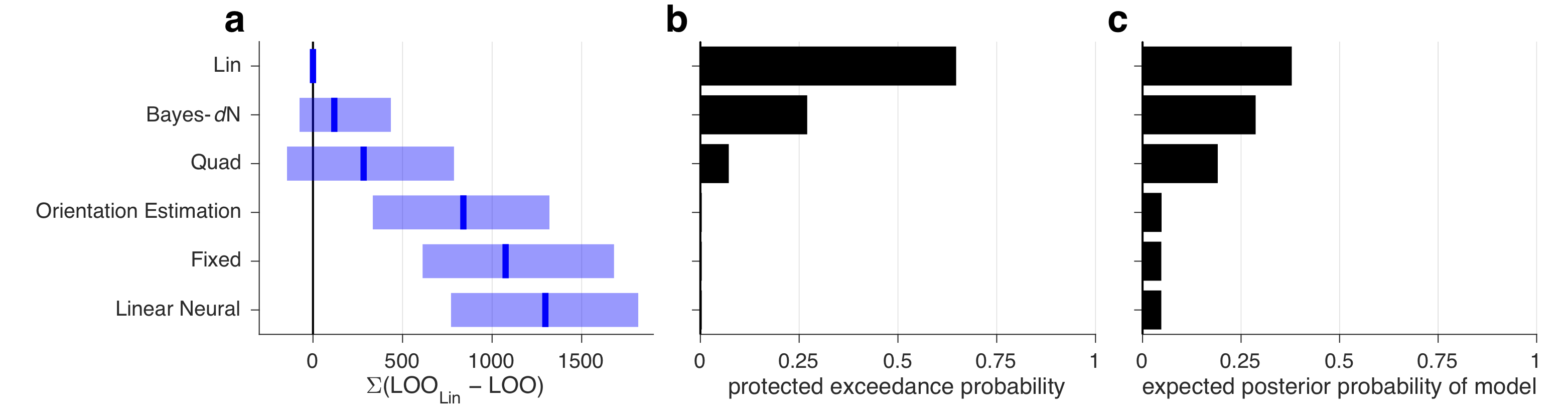

Supplement: S8 Fig — Models were fit to Task B category choices. See S1 Fig caption. (TIF) [file pcbi.1006572.s008.tif]

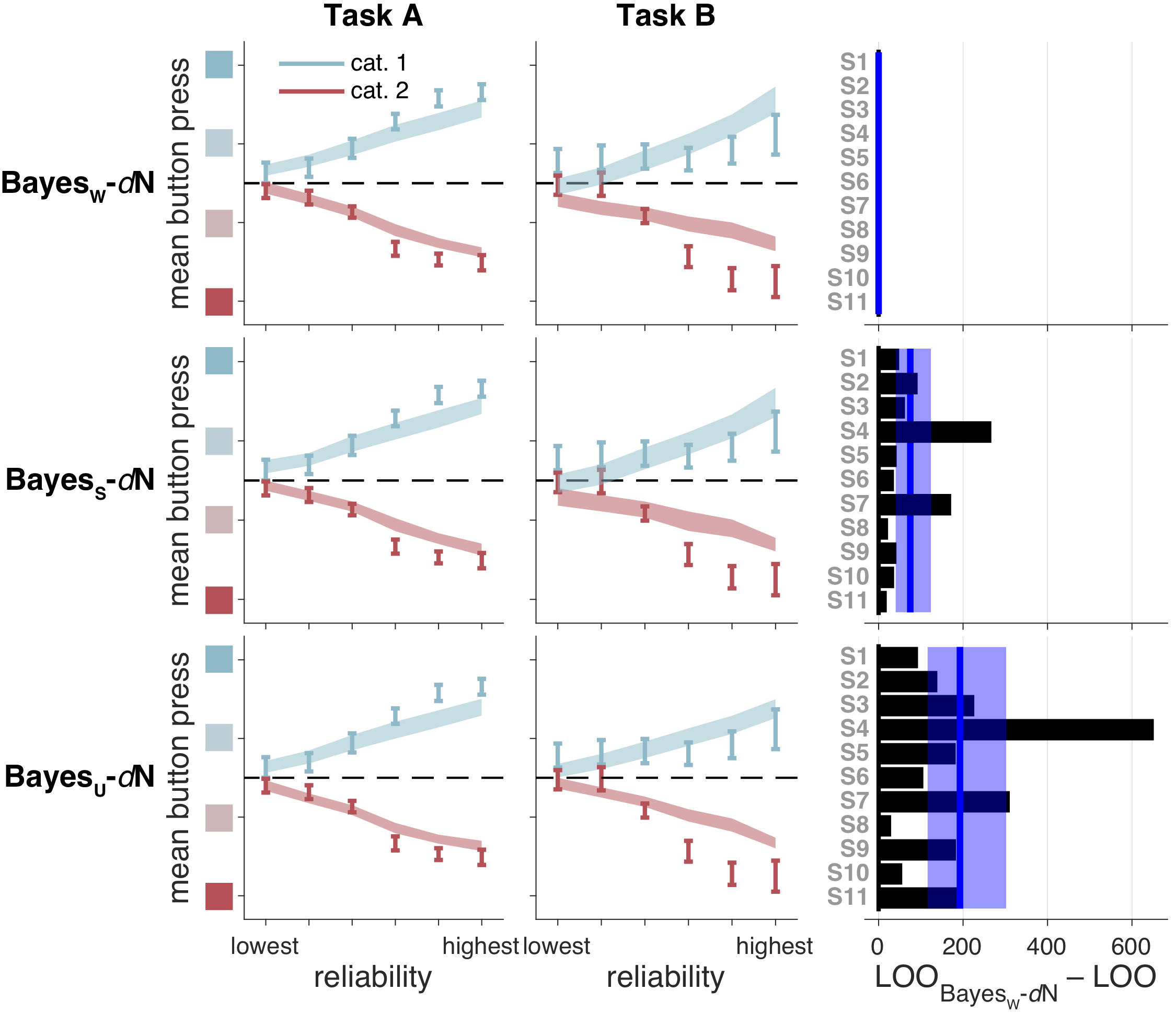

Supplement: S9 Fig — In the main text, BayesWeak-dN is referred to simply as Bayes. (TIF) [file pcbi.1006572.s009.tif]

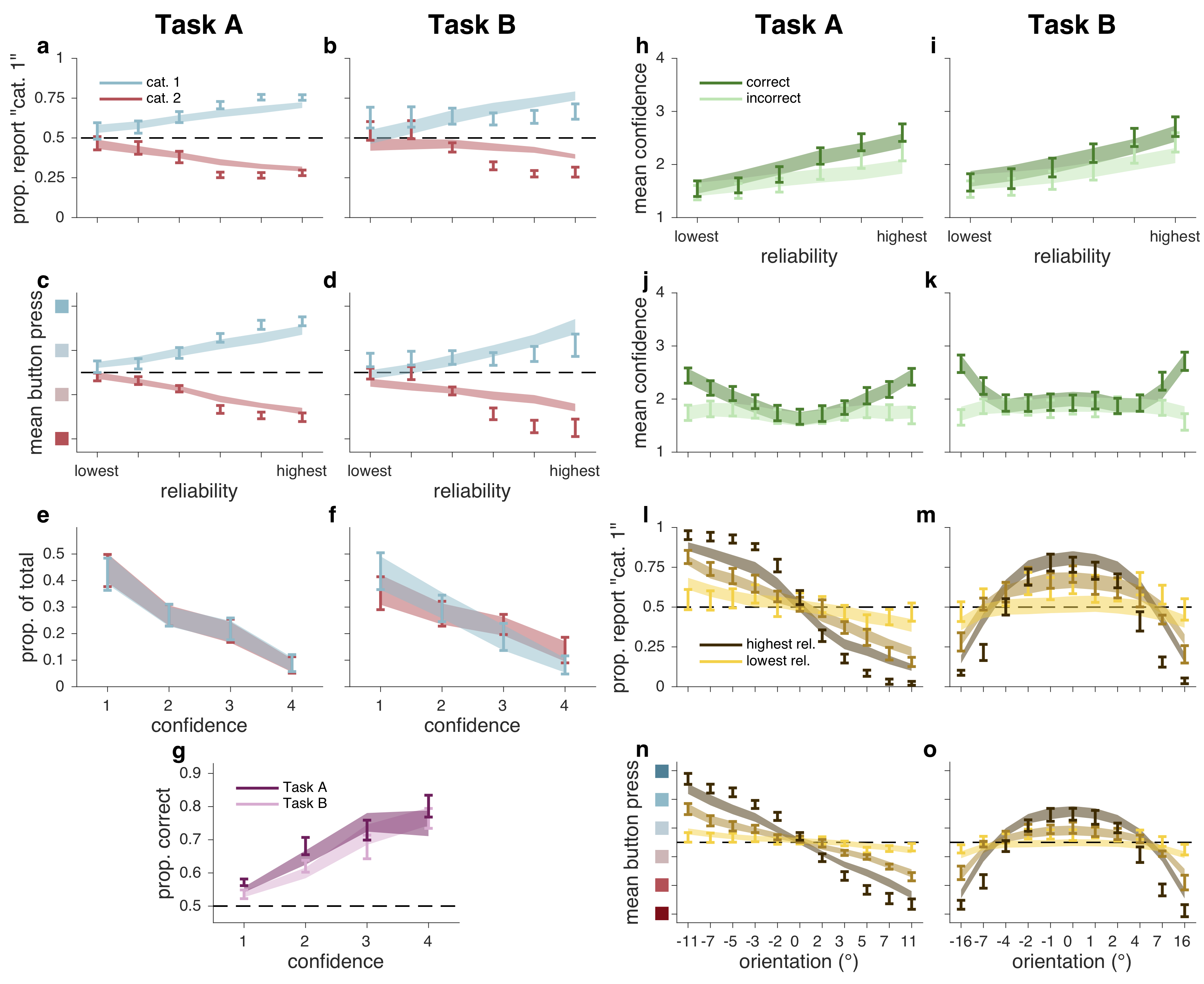

Supplement: S10 Fig — In the main text, BayesWeak-dN is referred to simply as Bayes. (TIF) [file pcbi.1006572.s010.tif]

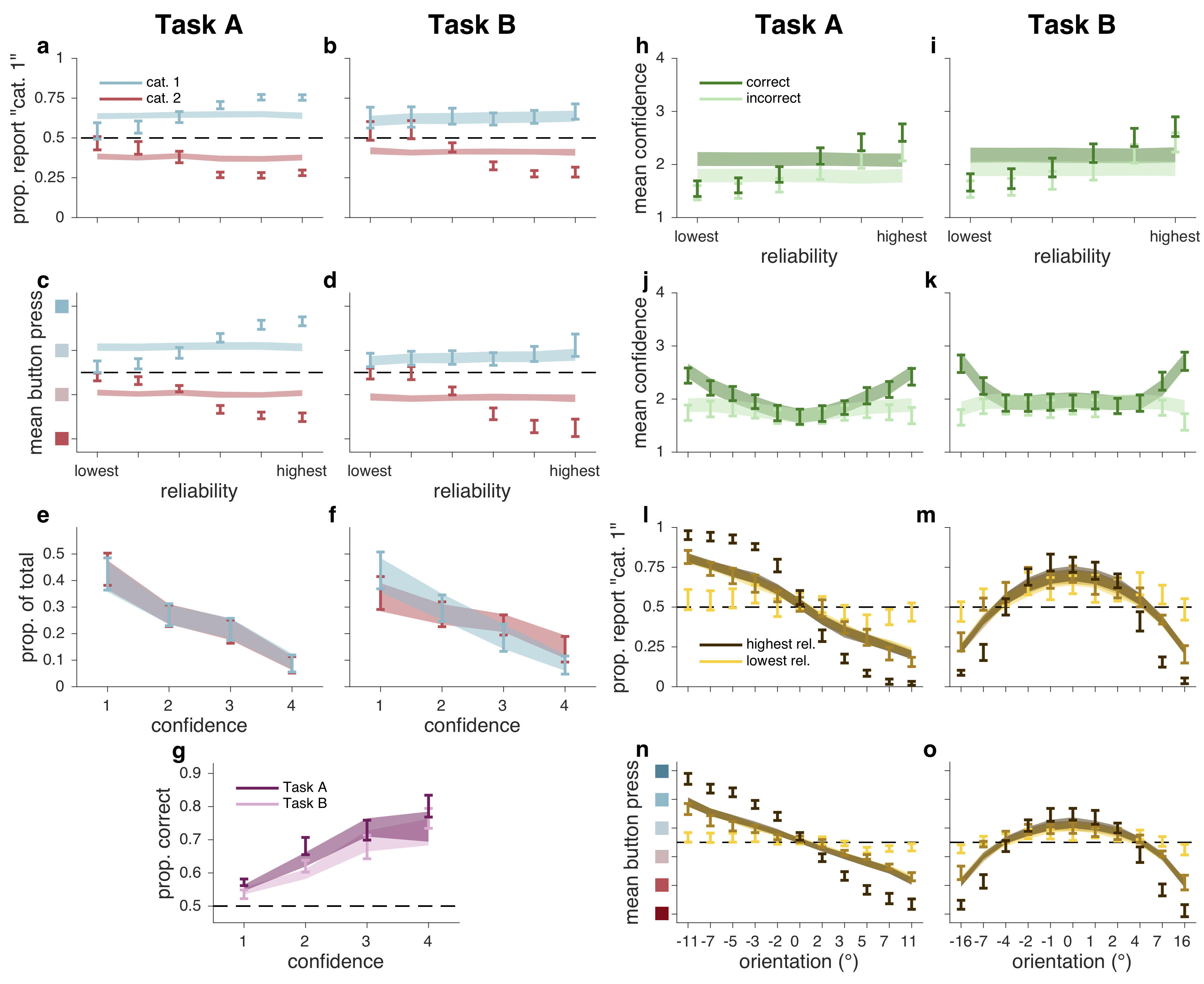

Supplement: S11 Fig — (TIF) [file pcbi.1006572.s011.tif]

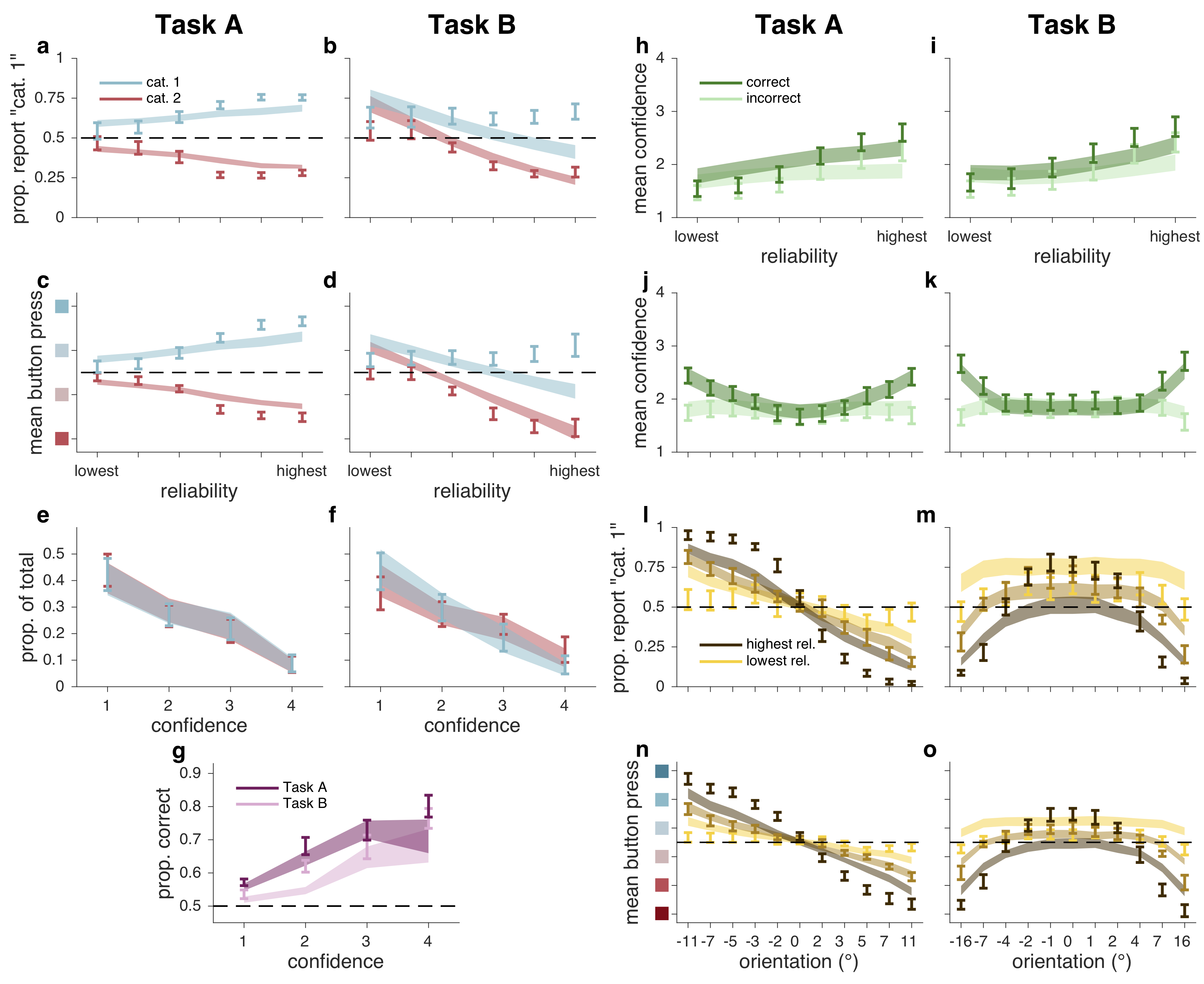

Supplement: S12 Fig — (TIF) [file pcbi.1006572.s012.tif]

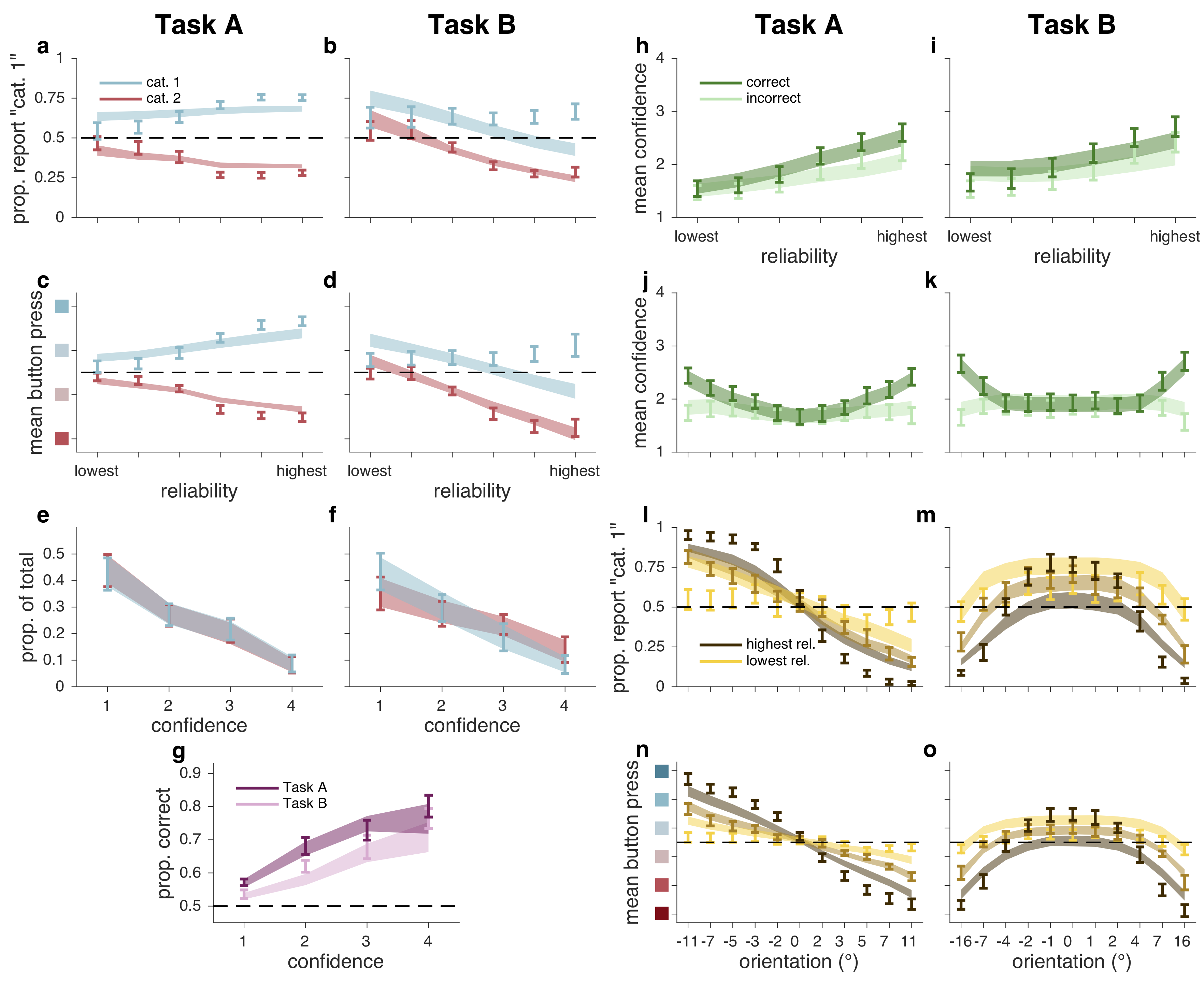

Supplement: S13 Fig — (TIF) [file pcbi.1006572.s013.tif]

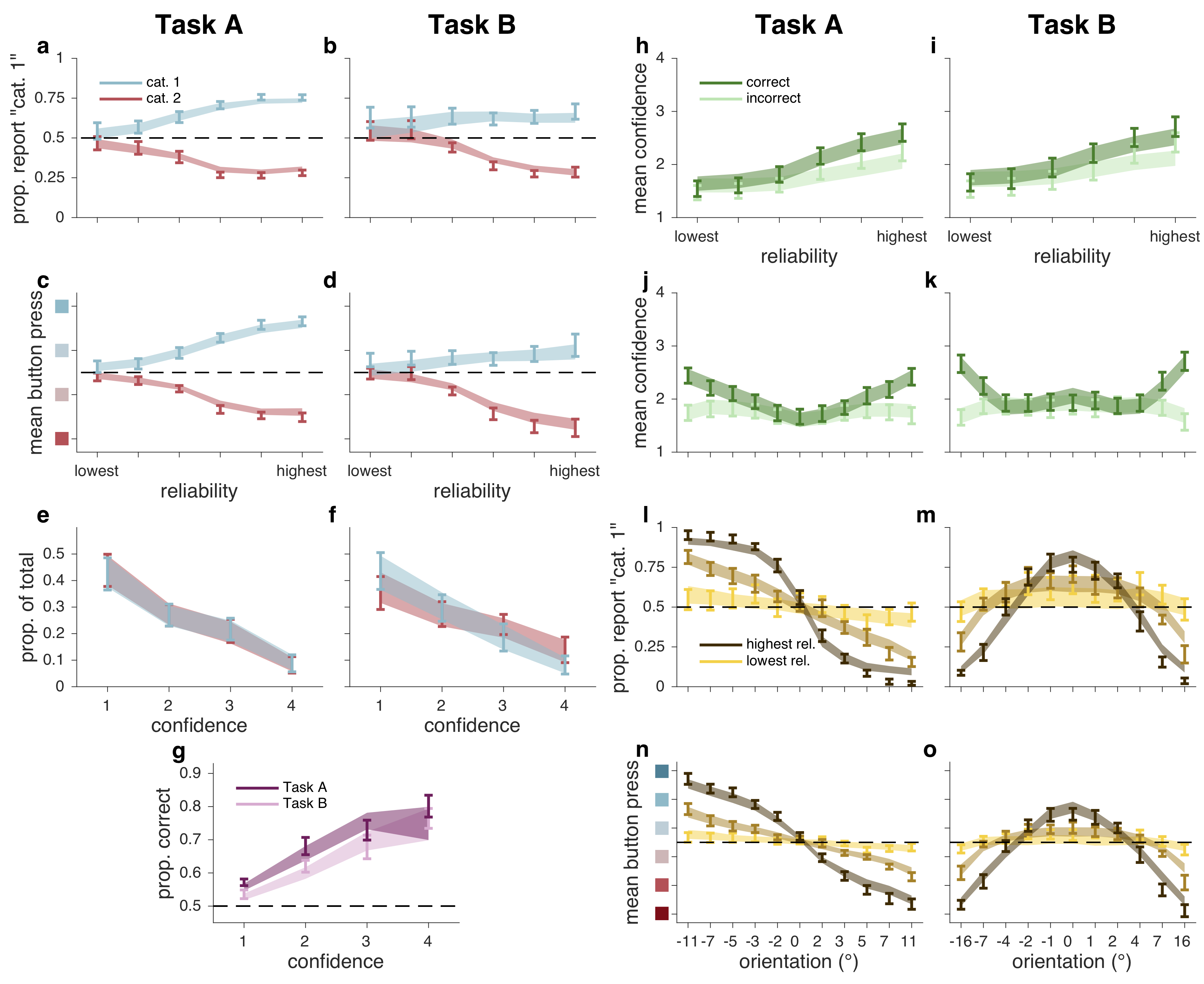

Supplement: S14 Fig — (TIF) [file pcbi.1006572.s014.tif]

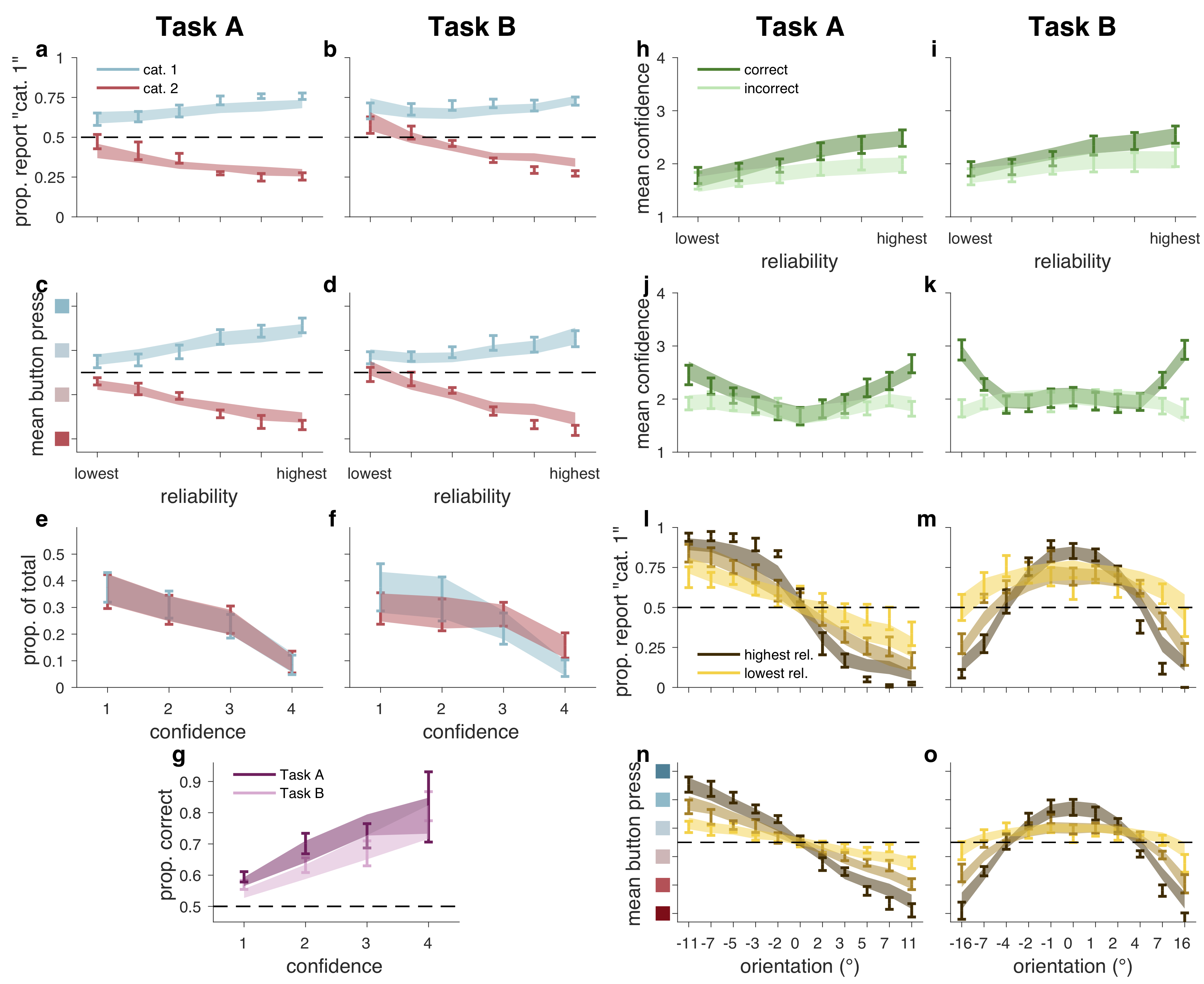

Supplement: S15 Fig — (TIF) [file pcbi.1006572.s015.tif]
