# Supplementary material for: Comparing Bayesian and non-Bayesian accounts of human confidence reports
Source: PLoS Comput Biol. 2018 Nov 13;14(11):e1006572. doi: 10.1371/journal.pcbi.1006572 (PMC6258566; doi:10.1371/journal.pcbi.1006572)
Supplement: S2 Table — See S1 Table caption. (PDF) [file pcbi.1006572.s017.pdf]

		12 pars. Fixed	13 pars. Bayes-dN	12 pars. Ori. Est.	13 pars. Lin. Neur.	16 pars. Lin
16 pars.	Quad	-3534 $[-4552, -2529]$	-581 $[-938, -278]$	-1241 $[-1798, -767]$	-270 $[-436, -117]$	-14 $[-325, 246]$
16 pars.	Lin	-3532 $[-4353, -2651]$	-572 $[-799, -339]$	-1232 $[-1566, -863]$	-259 $[-609, 124]$	
13 pars.	Lin. Neur.	-3255 $[-4343, -2231]$	-313 $[-724, 75]$	-972 $[-1599, -412]$		
12 pars.	Ori. Est.	-2302 $[-2881, -1705]$	651 $[425, 885]$			
13 pars.	Bayes-dN	-2956 $[-3723, -2163]$				
