# Supplementary material for: Comparing Bayesian and non-Bayesian accounts of human confidence reports
Source: PLoS Comput Biol. 2018 Nov 13;14(11):e1006572. doi: 10.1371/journal.pcbi.1006572 (PMC6258566; doi:10.1371/journal.pcbi.1006572)
Supplement: S3 Table — See S1 Table caption. (PDF) [file pcbi.1006572.s018.pdf]

		15 pars. Fixed	13 pars. Bayes_S-dN	16 pars. Bayes_W-dN	15 pars. Ori. Est.	16 pars. Lin. Neur.	22 pars. Lin
22 pars.	Quad	-3234 $[-4390, -2099]$	-1664 $[-2698, -958]$	-978 $[-1756, -406]$	-2156 $[-3352, -1192]$	-2060 $[-3368, -1037]$	-744 $[-1387, -224]$
22 pars.	Lin	-2480 $[-3323, -1645]$	-919 $[-1788, -279]$	-232 $[-900, 346]$	-1415 $[-2439, -439]$	-1326 $[-2442, -337]$	
16 pars.	Lin. Neur.	-1117 $[-2093, -349]$	421 $[-1095, 1689]$	1106 $[-374, 2583]$	-80 $[-222, 62]$		
15 pars.	Ori. Est.	-1043 $[-1962, -273]$	502 $[-934, 1693]$	1184 $[-202, 2588]$			
16 pars.	Bayes_W-dN	-2230 $[-3239, -1307]$	-691 $[-1082, -390]$				
13 pars.	Bayes_S-dN	-1534 $[-2425, -634]$					
