# Supplementary material for: Comparing Bayesian and non-Bayesian accounts of human confidence reports
Source: PLoS Comput Biol. 2018 Nov 13;14(11):e1006572. doi: 10.1371/journal.pcbi.1006572 (PMC6258566; doi:10.1371/journal.pcbi.1006572)
Supplement: S4 Table — See S1 Table caption. (PDF) [file pcbi.1006572.s019.pdf]

		8 pars. Fixed	9 pars. Bayes-dN	8 pars. Ori. Est.	9 pars. Lin. Neur.	10 pars. Lin
10 pars.	Quad	-1319 $[-2541, -611]$	-236 $[-1072, 358]$	-154 $[-772, 613]$	-729 $[-1365, -38]$	323 $[-423, 1127]$
10 pars.	Lin	-1690 $[-2534, -976]$	-595 $[-927, -311]$	-492 $[-1023, 238]$	-1087 $[-1690, -245]$	
9 pars.	Lin. Neur.	-591 $[-2068, 460]$	486 $[-504, 1211]$	591 $[406, 789]$		
8 pars.	Ori. Est.	-1190 $[-2614, -144]$	-114 $[-1026, 579]$			
9 pars.	Bayes-dN	-1095 $[-1657, -629]$				
