# Supplementary material for: Comparing Bayesian and non-Bayesian accounts of human confidence reports
Source: PLoS Comput Biol. 2018 Nov 13;14(11):e1006572. doi: 10.1371/journal.pcbi.1006572 (PMC6258566; doi:10.1371/journal.pcbi.1006572)
Supplement: S5 Table — See S1 Table caption. (PDF) [file pcbi.1006572.s020.pdf]

		15 pars. Fixed	13 pars. Bayes$_S$-dN	16 pars. Bayes$_W$-dN	15 pars. Ori. Est.	16 pars. Lin. Neur.	22 pars. Lin
22 pars.	Quad	−4367 [−6304, −2391]	−1670 [−3268, −39]	−1135 [−2501, 333]	−2836 [−4544, −1122]	−2449 [−4200, −969]	606 [6, 1269]
22 pars.	Lin	−5016 [−6727, −3090]	−2303 [−3578, −921]	−1773 [−2845, −451]	−3497 [−4860, −1817]	−3127 [−4549, −1575]	
16 pars.	Lin. Neur.	−1837 [−3566, −378]	846 [−609, 2092]	1386 [−13, 2732]	−345 [−933, 262]		
15 pars.	Ori. Est.	−1498 [−2877, −420]	1184 [23, 2129]	1724 [575, 2808]			
16 pars.	Bayes$_W$-dN	−3257 [−3965, −2494]	−533 [−920, −283]				
13 pars.	Bayes$_S$-dN	−2704 [−3351, −2027]					
