# Supplementary material for: Comparing Bayesian and non-Bayesian accounts of human confidence reports
Source: PLoS Comput Biol. 2018 Nov 13;14(11):e1006572. doi: 10.1371/journal.pcbi.1006572 (PMC6258566; doi:10.1371/journal.pcbi.1006572)
Supplement: S6 Table — See S1 Table caption. (PDF) [file pcbi.1006572.s021.pdf]

		19 pars.<br>Fixed	13 pars.<br>Bayes_U-dN	17 pars.<br>Bayes_S-dN	20 pars.<br>Bayes_W-dN	19 pars.<br>Ori. Est.	20 pars.<br>Lin. Neur.	30 pars.<br>Lin
30 pars.	Quad	-2014 $[-3036, -1186]$	-1096 $[-1807, -530]$	-523 $[-893, -220]$	-331 $[-562, -109]$	-1136 $[-1815, -638]$	-1124 $[-1922, -613]$	74 $[-195, 252]$
30 pars.	Lin	-2095 $[-2889, -1344]$	-1160 $[-1780, -694]$	-589 $[-841, -375]$	-396 $[-622, -186]$	-1218 $[-1680, -791]$	-1205 $[-1757, -785]$	
20 pars.	Lin. Neur.	-876 $[-1401, -395]$	55 $[-711, 693]$	623 $[99, 1184]$	801 $[253, 1491]$	-7 $[-219, 216]$		
19 pars.	Ori. Est.	-872 $[-1297, -487]$	56 $[-561, 574]$	620 $[218, 1089]$	813 $[356, 1394]$			
20 pars.	Bayes_W-dN	-1678 $[-2542, -1007]$	-767 $[-1262, -386]$	-190 $[-363, -82]$				
17 pars.	Bayes_S-dN	-1490 $[-2210, -886]$	-565 $[-1032, -266]$					
13 pars.	Bayes_U-dN	-907 $[-1572, -365]$						
