# Supplementary material for: Comparing Bayesian and non-Bayesian accounts of human confidence reports
Source: PLoS Comput Biol. 2018 Nov 13;14(11):e1006572. doi: 10.1371/journal.pcbi.1006572 (PMC6258566; doi:10.1371/journal.pcbi.1006572)
Supplement: S7 Table — See S1 Table caption. (PDF) [file pcbi.1006572.s022.pdf]

		15 pars. Fixed	13 pars. Bayes_S-dN	16 pars. Bayes_W-dN	15 pars. Ori. Est.	16 pars. Lin. Neur.	22 pars. Lin
22 pars.	Quad	-7326 [$-9955, -4905$]	-2833 [$-3807, -1926$]	-1361 [$-2022, -777$]	-7120 [$-9838, -4636$]	-6902 [$-10376, -3981$]	-1577 [$-2562, -750$]
22 pars.	Lin	-5759 [$-7866, -3694$]	-1240 [$-2567, 65$]	226 [$-812, 1246$]	-5530 [$-7707, -3539$]	-5337 [$-8191, -2846$]	
16 pars.	Lin. Neur.	-450 [$-1535, 1290$]	4114 [$733, 7796$]	5552 [$2338, 9135$]	-214 [$-1176, 1256$]		
15 pars.	Ori. Est.	-256 [$-841, 423$]	4311 [$1432, 7134$]	5727 [$3067, 8527$]			
16 pars.	Bayes_W-dN	-5967 [$-8702, -3369$]	-1454 [$-2179, -835$]				
13 pars.	Bayes_S-dN	-4505 [$-7282, -1816$]					
