# Supplementary material for: Comparing Bayesian and non-Bayesian accounts of human confidence reports
Source: PLoS Comput Biol. 2018 Nov 13;14(11):e1006572. doi: 10.1371/journal.pcbi.1006572 (PMC6258566; doi:10.1371/journal.pcbi.1006572)
Supplement: S8 Table — See S1 Table caption. (PDF) [file pcbi.1006572.s023.pdf]

		7 pars.<br>Fixed	8 pars.<br>Bayes-dN	7 pars.<br>Ori. Est.	8 pars.<br>Lin. Neur.	8 pars.<br>Lin
8 pars.	Quad	-777 $[-1361, -359]$	162 $[-290, 670]$	-531 $[-1059, -23]$	-988 $[-1526, -549]$	290 $[-138, 793]$
8 pars.	Lin	-1084 $[-1675, -619]$	-117 $[-436, 76]$	-830 $[-1317, -334]$	-1294 $[-1825, -778]$	
8 pars.	Lin. Neur.	215 $[-566, 827]$	1174 $[535, 1772]$	457 $[254, 685]$		
7 pars.	Ori. Est.	-255 $[-987, 369]$	707 $[119, 1259]$			
8 pars.	Bayes-dN	-964 $[-1290, -663]$				
